# Oral Health Literacy and Preventive Behaviors Among Caregivers of Dependent Older Adults in Sisaket Province, Thailand: A Structural Equation Modeling Study

**DOI:** 10.3390/ijerph23040451

**Published:** 2026-04-01

**Authors:** Kunlachart Wattanavong Valuvanaluk, Aree Butsorn, Putthikrai Pramual

**Affiliations:** 1 College of Medicine and Public Health, Ubon Ratchathani University, Ubon Ratchathani 34190, Thailand; kunlachartwatanawong.va.66@ubu.ac.th; 2 Centers for Disease Control and Prevention, Sisaket Provincial Health Office, Sisaket 33000, Thailand; putthikrai.pramual@gmail.com

**Keywords:** oral health literacy, caregivers, dependent older adults, gingivitis, preventive behaviors, structural equation modeling, health belief model, social support, Sisaket Province, Thailand

## Abstract

**Highlights:**

**Public health relevance—How does this work relate to a public health issue?**

- Dependent older adults face a high burden of preventable oral diseases, particularly gingivitis, due to reliance on caregivers for daily oral care in ageing societies.
- In Sisaket Province, Thailand, gaps in caregivers’ oral health literacy and behavioral capacity contribute to inadequate preventive practices and increased oral health risks.

**Public health significance—Why is this work of significance to public health?**

- This study develops a theory-driven structural equation model integrating Health Belief Model and Social Cognitive Theory to explain caregiver-driven oral health behaviors.
- The model explains a substantial proportion of behavioral variance (68.3%), highlighting the critical roles of attitude, self-efficacy, and social support beyond knowledge alone.

**Public health implications—What are the key implications or messages for practitioners, policy makers and/or researchers in public health?**

- Oral health interventions should move beyond knowledge-based education to include skill-building, self-efficacy enhancement, and structured social support for caregivers.
- Integrating caregiver-focused oral health literacy programs into primary care and community-based long-term care systems can serve as a scalable strategy to improve oral health outcomes in ageing populations.

**Abstract:**

Background: Dependent older adults experience a high burden of preventable oral conditions, particularly gingivitis, while caregivers’ preventive practices remain insufficiently explained by conventional single-factor models. This study aimed to develop and validate a theory-informed structural equation model (SEM) to explain how oral health literacy and psychosocial determinants shape preventive behaviors for gingivitis among caregivers of dependent older adults in Sisaket Province, Thailand. Methods: A cross-sectional analytical study was conducted among 420 caregivers selected using multistage random sampling. Data were collected using a validated questionnaire based on the Health Belief Model and Social Cognitive Theory, covering knowledge, attitude, perceived severity, perceived benefits, self-efficacy, preventive behaviors, and social support. Reliability was excellent. Descriptive statistics, Pearson’s correlation, and SEM with maximum likelihood estimation were applied. Results: The SEM demonstrated excellent fit (χ^2^/df = 0.80, *p* = 1.000; CFI = 1.000; TLI = 1.008; RMSEA = 0.000; SRMR = 0.092). Knowledge directly influenced attitude (β = 0.355, *p* = 0.013) and self-efficacy (β = 0.381, *p* = 0.003). Attitude (β = 0.406, *p* < 0.001), self-efficacy (β = 0.384, *p* < 0.001), and social support (β = 0.260, *p* < 0.001) were significant predictors of preventive behaviors. The model explained 68.3% of variance in behaviors, with knowledge exerting significant total effects through mediated pathways (β_total = 0.290, *p* < 0.001). Conclusions: A theory-based SEM effectively explains caregiver preventive oral health behaviors in a rural, resource-limited setting. Interventions should integrate literacy-focused education with strategies that strengthen attitudes, self-efficacy, and social support to achieve sustainable improvements in dependent older adults’ oral health.

## 1. Introduction

Population ageing is accelerating globally and is reshaping public health priorities. The World Health Organization projects that the number of adults aged ≥ 60 years will increase from 1 billion in 2020 to 2 billion by 2050 [1]. Thailand is experiencing rapid demographic transition; older adults accounted for approximately 19% of the population in 2022 and are projected to reach 28% by 2040 [2]. Sisaket Province in northeastern Thailand has already entered an aged society, with older adults constituting about 22% of the provincial population [3]. This demographic shift intensifies the need to address preventable conditions that undermine healthy ageing.

Oral health is closely linked to nutrition, systemic health, and quality of life in older adults [4,5]. Periodontal diseases, including gingivitis, are among the most prevalent oral conditions worldwide, with estimates frequently exceeding 60% in older populations [6,7]. Thai national survey evidence similarly indicates a high burden of periodontal conditions among older adults [8]. The situation is often worse among dependent older adults who require assistance with activities of daily living. Physical limitations, cognitive impairment, multimorbidity, and reliance on caregivers can compromise daily oral hygiene, accelerate plaque accumulation, and increase gingival inflammation and progression to periodontitis [9]. These oral conditions may contribute to broader systemic complications such as cardiovascular disease, worsened glycemic control, respiratory infections, and malnutrition [10,11].

Caregivers play a central role in the daily oral hygiene of dependent older adults; however, many caregivers lack adequate oral health literacy—the knowledge and skills required to make informed oral health decisions and implement preventive behaviors consistently [12,13,14]. Importantly, knowledge alone often fails to translate into sustained behavioral change, particularly in resource-limited contexts where caregivers face competing priorities and constraints. Prior interventions have frequently emphasized didactic knowledge transfer while under-addressing psychosocial determinants such as attitudes, perceived disease severity and benefits, self-efficacy, and social support—factors consistently shown to influence preventive behaviors [15,16,17].

To address these gaps, this study developed and validated a comprehensive SEM grounded in the Health Belief Model (HBM) and Social Cognitive Theory (SCT). HBM explains how perceived severity and perceived benefits shape health-related attitudes and decisions [18], while SCT highlights self-efficacy and social influences as key drivers of behavior. SEM is particularly appropriate because it enables simultaneous testing of direct and indirect pathways among multiple interrelated determinants, offering a more complete understanding than single-equation approaches [19].

This study had the following objectives: (1) assess levels of oral health literacy and preventive behaviors among caregivers of dependent older adults in Sisaket Province; (2) examine relationships among knowledge, attitude, perceived severity, perceived benefits, self-efficacy, social support, and preventive behaviors; and (3) develop and validate an SEM explaining these relationships. We hypothesized that oral health literacy (operationalized as knowledge) would influence preventive behaviors directly and indirectly through attitudes and self-efficacy, and that social support would contribute to behavior both directly and via psychosocial pathways.

## 2. Materials and Methods

### 2.1. Study Design and Setting

A cross-sectional analytical study was conducted in Sisaket Province, northeastern Thailand, from September to November 2023. Sisaket is predominantly rural and agricultural, with approximately 1,464,025 residents, of whom about 22% are aged ≥ 60 years [3].

### 2.2. Study Population

The target population comprised primary caregivers of dependent older adults residing in Sisaket Province. Dependent older adults were defined as individuals aged ≥ 60 years requiring assistance with activities of daily living. Provincial registration data indicated approximately 32,000 dependent older adults in 2023 [3].

### 2.3. Sample Size

Sample size was determined using Kline’s recommendation for SEM (10–20 participants per estimated parameter) [19]. With 21 parameters in the proposed model, a minimum of 210–420 participants was required. To ensure adequate power and account for potential incomplete responses, 420 caregivers were recruited (20:1 ratio).

### 2.4. Sampling Procedure

Multistage random sampling was applied:

Stage 1: 6 districts were selected from 22 districts using probability proportional to size (PPS) based on the number of registered dependent older adults.

Stage 2: Sub-districts within selected districts were chosen using PPS.

Stage 3: Dependent older adults and their caregivers were identified through community health volunteer networks and sub-district health-promoting hospital records.

Stage 4: Eligible caregiver–older adult dyads were randomly selected until the required sample size was achieved, proportionally allocated across selected sub-districts.

### 2.5. Eligibility Criteria

Inclusion criteria: caregivers aged ≥ 18 years; primary caregiver providing most daily care; caregiving duration ≥ 6 months; able to communicate in Thai; willing to participate and provide consent.

Exclusion criteria: professional paid healthcare workers serving as caregivers; severe mental health conditions impairing judgment; temporary/secondary caregivers.

### 2.6. Research Instrument and Measures

A structured questionnaire was developed based on the HBM and literature review [17,20,21]. It comprised eight sections:

(1) Demographics (age, sex, education, occupation, income, relationship to older adult, caregiving duration, caregiver health status). (2) Oral health knowledge (3) Attitude (4) Perceived severity (5) Perceived benefits (6) Self-efficacy (7) Preventive behaviors for gingivitis (8) Social support

All attitudinal and behavioral constructs were measured using Likert-type response formats, with higher scores indicating more favorable beliefs, stronger perceived capability, and better preventive practices. Composite scores were calculated for each construct and treated as continuous variables in subsequent analyses. The questionnaire was developed in Thai and underwent a rigorous content validation process. Three experts in dental public health, geriatric care, and behavioral science independently reviewed the instrument to ensure relevance, clarity, and cultural appropriateness for caregivers of dependent older adults in rural Thailand. Minor wording revisions were made based on their feedback to improve comprehension and contextual suitability. A pilot study was conducted among caregivers from a community with similar characteristics to the study area. The pilot test assessed item clarity, completion time, and internal consistency of the scales. Reliability testing showed acceptable to high internal consistency across constructs, supporting the suitability of the instrument for field implementation. To further evaluate construct validity, confirmatory factor analysis (CFA) was performed as part of the structural equation modeling procedure. Factor loadings were examined to verify that observed variables adequately represented their latent constructs, and reliability indices such as composite reliability and average variance extracted were assessed. These procedures confirmed that the measurement model demonstrated satisfactory psychometric properties and was appropriate for testing the hypothesized structural relationships. Data were collected through face-to-face interviews conducted by trained research assistants and community health volunteers. This approach was chosen to minimize missing responses and to ensure that caregivers with limited literacy could fully participate. Standardized instructions were used to maintain consistency across interviewers.

### 2.7. Validity and Reliability

Content validity was assessed by three experts (public health dentist, community health nursing expert, behavioral science researcher). Pilot testing was conducted among 30 caregivers with similar characteristics in another province. Internal consistency reliability was excellent.

### 2.8. Data Collection

Face-to-face interviews were conducted by trained research assistants after a two-day training on ethics, interview techniques, and questionnaire administration. Interviews lasted 45–60 min and took place at participants’ homes or locations convenient to participants. Completed questionnaires were checked daily by the principal investigator, and missing/inconsistent responses were followed up within 48 h.

### 2.9. Statistical Analysis

Data were analyzed using JAMOVI (version 2.6.44) for structural equation modeling (SEM). Descriptive statistics included frequencies, percentages, means, and standard deviations. Pearson’s correlation examined bivariate relationships. SEM used maximum likelihood estimation with robust standard errors (MLR) where appropriate. Model fit was evaluated with χ^2^ and χ^2^/df (≤3.0), CFI (≥0.95), TLI (≥0.95), RMSEA (≤0.06), SRMR (≤0.08), and PNFI (≥0.50). Direct and indirect effects were estimated; significance level was α = 0.05.

### 2.10. Ethical Considerations

This study was approved by the Human Research Ethics Committee of Ubon Ratchathani University (Protocol No. UBU-REC-110/2568; approved on 15 September 2025). All participants provided written informed consent. Confidentiality was ensured via anonymous coding and secure data storage with access restricted to the research team.

## 3. Results

### 3.1. Participant Characteristics

A total of 420 caregivers participated. Most were female (82.1%). The predominant age group was 50–59 years (39.8%), followed by 40–49 years (32.4%), ≥60 years (18.6%), and <40 years (9.3%). Most caregivers were farmers (95.7%). Monthly household income was largely < 5000 THB (75.0%). Educational attainment was primarily upper secondary/vocational (52.1%) and primary education (39.3%). Approximately 24.8% reported chronic conditions, mainly hypertension (22.1%) and diabetes (2.6%). The general characteristics of the participants are presented in Table 1.

### 3.2. Levels of Study Variables

Attitude toward oral health showed the highest mean score (*M* = 39.40, *SD* = 5.35), followed by perceived benefits (*M* = 25.10, *SD* = 3.51) and perceived severity (*M* = 24.80, *SD* = 3.60), all of which were interpreted at a high level. These findings suggest that caregivers generally recognized the importance of oral health problems and the benefits of preventive care. In contrast, knowledge (*M* = 6.59, *SD* = 1.40), self-efficacy (*M* = 23.50, *SD* = 4.98), preventive oral health behaviors (*M* = 23.10, *SD* = 4.46), and social support (*M* = 23.30, *SD* = 4.49) were at a moderate level. This implies that although caregivers possessed favorable attitudes and perceived risks and benefits, their actual knowledge, confidence in performing care, and preventive practices were not yet at a high level. Overall, the pattern of findings indicates a gap between cognitive–perceptual factors (attitude and beliefs) and practical behavioral capacity (knowledge, self-efficacy, and preventive practices), highlighting the need for interventions aimed at strengthening caregivers’ skills, confidence, and support systems to improve preventive oral health behaviors. The levels of study variables are summarized in Table 2.

### 3.3. Reliability and Convergent Validity

Composite reliability (CR) values ranged from 0.664 to 0.913. Most constructs exceeded the recommended threshold of 0.70, indicating satisfactory internal consistency. In particular, self-efficacy (CR = 0.913), preventive behaviors (CR = 0.900), and social support (CR = 0.887) demonstrated high reliability, reflecting strong coherence among their respective indicators. Although the CR value for knowledge (CR = 0.664) was slightly below the conventional criterion, it remained within an acceptable range for exploratory behavioral research. Regarding convergent validity, AVE values ranged from 0.425 to 0.665. The majority of constructs—including attitude (AVE = 0.582), perceived benefits (AVE = 0.562), self-efficacy (AVE = 0.665), social support (AVE = 0.561), and preventive behaviors (AVE = 0.614)—exceeded the recommended threshold of 0.50, indicating that these constructs adequately captured the variance of their indicators. However, knowledge (AVE = 0.425) and perceived severity (AVE = 0.441) showed slightly lower AVE values. Despite this, both constructs were retained due to their acceptable composite reliability and theoretical importance within the Health Belief Model framework. This approach is consistent with methodological recommendations suggesting that AVE values below 0.50 may be tolerated when CR exceeds 0.60. Overall, these findings confirm that the measurement model demonstrates satisfactory reliability and convergent validity, supporting the adequacy of the constructs for subsequent structural equation modeling. The reliability and convergent validity of the constructs are presented in Table 3. 

### 3.4. Structural Equation Model Fit

The hypothesized SEM showed very good model fit: χ^2^/df = 0.80, *p* = 0.288; CFI = 1.000; TLI = 1.008; RMSEA = 0.006 (90% CI 0.000–0.013). The SRMR was 0.108, which is slightly above the conventional cut-off, but still considered acceptable given the model complexity and large number of indicators. Other indices also supported good model fit (PNFI = 0.926; NFI = 0.960; IFI = 1.008). The model fit indices are shown in Table 4.

### 3.5. Path Coefficients and Effects

The structural equation model showed that preventive oral health behaviors among caregivers were influenced by a network of cognitive, motivational, and social factors. Knowledge had significant direct effects on both attitude (β = 0.355, *p* = 0.013) and self-efficacy (β = 0.381, *p* = 0.003), indicating that greater understanding of oral health contributed to improved motivation and confidence in caregiving practices. Perceived severity (β = 0.332, *p* < 0.001) and perceived benefits (β = 0.328, *p* < 0.001) also significantly predicted attitude, confirming the role of health beliefs in shaping caregivers’ behavioral readiness. Social support showed a significant positive effect on self-efficacy (β = 0.317, *p* < 0.001), highlighting the importance of interpersonal and community support systems.

Regarding behavioral outcomes, attitude (β = 0.406, *p* < 0.001) exerted the strongest direct influence on preventive oral health behaviors, followed by self-efficacy (β = 0.384, *p* < 0.001) and social support (β = 0.260, *p* < 0.001). In addition to these direct effects, knowledge, perceived severity, and perceived benefits demonstrated indirect influences on preventive behaviors through attitude, while knowledge and social support also affected behavior indirectly through self-efficacy. These findings indicate that preventive behaviors are shaped primarily through mediating motivational mechanisms rather than direct cognitive effects alone.

**Figure 1:**
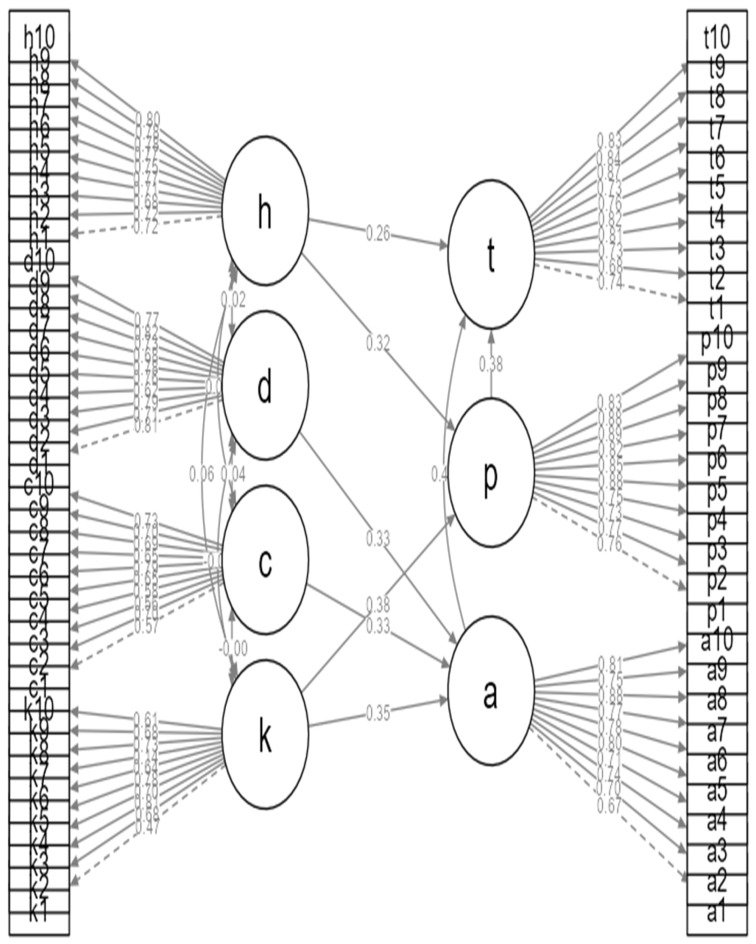
Structural Equation Model of Oral Health Literacy and Preventive Behaviors among Caregivers of Dependent Older Adults. The model illustrates the relationships among latent constructs, including knowledge (K), perceived severity (C), perceived benefits (D), social support (H), attitude (A), self-efficacy (P), and preventive behaviors (T). Standardized path coefficients (β) are presented along directional arrows. Observed indicators for each latent construct are shown as rectangular variables. Solid arrows represent significant direct effects, while dashed lines indicate measurement relationships between latent variables and their observed indicators.

The model explained substantial variance in key endogenous constructs. Attitude was jointly explained by knowledge and health beliefs, self-efficacy by knowledge and social support, and preventive behaviors by attitude, self-efficacy, and social support. Overall, the SEM (Figure 1) results support the theoretical assumptions of the Health Belief Model, demonstrating that preventive oral health behaviors among caregivers arise from an integrated pathway linking knowledge and beliefs to motivation, confidence, and supportive environments. The path coefficients of the structural model are presented in Table 5.

## 4. Discussion

### 4.1. Principal Findings

This study developed and validated a theory-informed SEM explaining caregiver preventive behaviors for gingivitis among dependent older adults in Sisaket Province, Thailand. The model fit the empirical data extremely well and explained a substantial proportion of variance in preventive behaviors. Findings support combined use of HBM and SCT to understand preventive behavior in the unique “proxy care” context where caregivers perform or supervise oral health practices for dependent individuals.

### 4.2. Knowledge as a Foundational Determinant via Mediated Pathways

Knowledge significantly influenced both attitude and self-efficacy, but its primary contribution to preventive behaviors was indirect. This aligns with broader health behavior literature describing the knowledge–behavior gap, wherein information alone is insufficient for sustained behavior change [22]. Practically, caregiver education should be designed to go beyond didactic delivery and incorporate skill-building components and supportive feedback to translate knowledge into confident, routine action.

### 4.3. Attitude as a Central Mediator

Attitude emerged as the strongest direct predictor of preventive behaviors and was shaped by knowledge, perceived severity, perceived benefits, and social support. This pattern reinforces HBM mechanisms, indicating that perceived threat and perceived benefits underpin motivational readiness, which then drives behavior [18]. In settings where oral health may be underprioritized, emphasizing the consequences of gingivitis and the tangible gains of preventive actions can shift attitudes and strengthen adherence.

### 4.4. Self-Efficacy and Social Support as Action Enablers

Self-efficacy had a robust effect on behaviors, consistent with SCT’s emphasis on confidence as a driver of performance under constraints [18]. Social support influenced behaviors directly and indirectly, suggesting that caregivers benefit from both practical support (resources, facilitation) and normative/informational support that strengthens attitudes. In rural communities, support may derive from family networks, community health volunteers, and primary care teams. Interventions that institutionalize caregiver support mechanisms may sustain behaviors beyond short-term education.

### 4.5. Strengths and Limitations

Strengths include a large randomly sampled cohort, validated measures with excellent reliability, and a theory-based SEM enabling estimation of complex pathways. Limitations include cross-sectional design limiting causal inference, self-reported measures susceptible to bias, and generalizability constrained to rural Sisaket. Future research should consider longitudinal designs, objective behavioral assessment, and inclusion of additional determinants (caregiver burden, service access, older adult characteristics).

### 4.6. Public Health Implications (IJERPH-Focused)

These findings have implications for healthy ageing and community-based long-term care. As informal caregivers increasingly deliver frontline care within households, strengthening caregiver health literacy and psychosocial capacity represents a scalable public health strategy in low-resource settings. Integrating oral health promotion into primary care, community health volunteer systems, and long-term care services may reduce preventable oral disease burden and associated downstream systemic complications. Programs should combine caregiver education with mastery-based training to enhance self-efficacy, and structured support (peer groups, family involvement, home-visit reinforcement) to sustain behaviors.

## 5. Conclusions

This study provides robust empirical evidence that a theory-driven structural equation model (SEM), grounded in the Health Belief Model (HBM) and Social Cognitive Theory (SCT), can effectively explain preventive oral health behaviors among caregivers of dependent older adults in rural Thailand. The findings highlight that caregiver behavior is shaped through complex and interrelated pathways rather than direct linear effects.

Specifically, knowledge did not exert a strong direct influence on preventive behaviors; instead, its effect was primarily mediated through attitudinal change and self-efficacy. This finding reinforces the well-documented “knowledge–behavior gap” in health promotion research and underscores the importance of addressing cognitive and motivational mechanisms beyond information provision alone. Caregivers who developed positive attitudes toward oral health and greater confidence in their caregiving abilities were significantly more likely to engage in consistent preventive practices.

In addition, social support emerged as a critical determinant, influencing preventive behaviors both directly and indirectly through self-efficacy. This emphasizes the importance of contextual and environmental factors, particularly in rural and community-based settings where caregiving is embedded within family and social networks. The integration of interpersonal and community-level support systems is therefore essential for sustaining behavior change.

From a practical perspective, the findings suggest that oral health promotion programs targeting caregivers should adopt a comprehensive and multi-component approach. Interventions should not only enhance caregiver knowledge but also actively promote positive attitudes, strengthen self-efficacy through hands-on training and skill development, and foster supportive social environments involving family members, community health volunteers, and primary healthcare providers. Such integrated strategies are more likely to produce sustainable improvements in gingivitis prevention and overall oral health outcomes among dependent older adults.

At the policy level, these results support the integration of caregiver-focused oral health literacy interventions into community-based primary care and long-term care systems. Strengthening caregiver capacity represents a scalable and cost-effective strategy for addressing oral health disparities in ageing populations, particularly in low-resource rural settings.

Despite its contributions, this study has some limitations. The cross-sectional design limits causal inference, and reliance on self-reported measures may introduce reporting bias. Future research should employ longitudinal or experimental designs to validate causal pathways and examine the long-term effectiveness of intervention models derived from this framework.

In conclusion, this study advances the understanding of caregiver-mediated oral health behaviors by demonstrating the importance of psychosocial and contextual mechanisms. The proposed SEM offers a theoretically grounded and empirically supported framework that can inform the design of effective oral health promotion strategies for dependent older adults.

## Figures and Tables

**Table 1:** General Characteristics of the Participants (*n* = 420).

General Information	Number (*n*)	Percentage (%)
Sex		
Male	75	17.9
Female	345	82.1
Age		
<40 years	39	9.3
40–49 years	136	32.4
50–59 years	167	39.8
≥60 years	78	18.6
Occupation		
Farmer	402	95.7
Government officer/State enterprise employee	0	0.0
Private company employee	0	0.0
Self-employed	18	4.3
Other	0	0.0
Monthly Income		
<5000 Baht	315	75.0
5001–10,000 Baht	97	23.1
10,001–15,000 Baht	7	1.7
>15,000 Baht	1	0.2
Education Level		
No formal education	0	0.0
Primary education	165	39.3
Lower secondary education	36	8.6
Upper secondary education/Vocational certificate	219	52.1
Diploma/High vocational certificate	0	0.0
Bachelor’s degree or higher	0	0.0
Underlying Disease		
None	316	75.2
Present	104	24.8

**Table 2:** Mean Scores and Standard Deviations of Variables in the Study (*n* = 420).

Variables	Mean	Standard Deviation (*SD*)	Interpretation Level
Knowledge	6.59	1.40	Moderate
Attitude	39.40	5.35	High
Perceived Severity	24.80	3.60	High
Perceived Benefits	25.10	3.51	High
Self-Efficacy	23.50	4.98	Moderate
Preventive Behaviors	23.10	4.46	Moderate
Social Support	23.30	4.49	Moderate

**Table 3:** Composite Reliability (CR) and Convergent Validity (AVE) of Latent Constructs in the Measurement Model.

Construct	ω (CR)	AVE	Interpretation
Knowledge (K)	0.664	0.425	Acceptable
Attitude (A)	0.876	0.582	Good
Perceived Severity (C)	0.799	0.441	Acceptable
Perceived Benefits (D)	0.814	0.562	Good
Self-Efficacy (P)	0.913	0.665	Excellent
Social Support (H)	0.887	0.561	Good
Preventive Behaviors (T)	0.900	0.614	Good

**Table 4:** User model versus baseline model.

	Model
Comparative Fit Index (CFI)	1.000
Tucker–Lewis Index (TLI)	1.008
Bentler-Bonett Non-normed Fit Index (NNFI)	1.008
Relative Noncentrality Index (RNI)	1.008
Bentler-Bonett Normed Fit Index (NFI)	0.960
Bollen’s Relative Fit Index (RFI)	0.958
Bollen’s Incremental Fit Index (IFI)	1.008
Parsimony Normed Fit Index (PNFI)	0.926

**Table 5:** Path Coefficients of the Structural Model.

Relationship	Estimate	β	z	*p*-Value	Interpretation
Knowledge (K) → Attitude (A)	0.510	0.355	2.49	0.013	Statistically significant
Perceived Severity (C) → Attitude (A)	0.391	0.332	4.58	<0.001	Statistically significant
Perceived Benefits (D) → Attitude (A)	0.270	0.328	5.36	<0.001	Statistically significant
Knowledge (K) → Self-Efficacy (P)	0.625	0.381	2.92	0.003	Statistically significant
Social Support (H) → Self-Efficacy (P)	0.338	0.317	5.56	<0.001	Statistically significant
Attitude (A) → Preventive Behaviors (T)	0.448	0.406	8.30	<0.001	Statistically significant
Self-Efficacy (P) → Preventive Behaviors (T)	0.371	0.384	7.47	<0.001	Statistically significant
Social Support (H) → Preventive Behaviors (T)	0.268	0.260	5.27	<0.001	Statistically significant

## Data Availability

The datasets generated and analyzed during the current study are not publicly available due to ethical restrictions and participant privacy protection but are available from the corresponding author upon reasonable request and with permission from the Human Research Ethics Committee.
